# Development of the General Parenting Observational Scale to assess parenting during family meals

**DOI:** 10.1186/s12966-015-0207-3

**Published:** 2015-04-10

**Authors:** Kyung E Rhee, Susan Dickstein, Elissa Jelalian, Kerri Boutelle, Ronald Seifer, Rena Wing

**Affiliations:** Department of Pediatrics, School of Medicine, University of California, San Diego, La Jolla, USA; Department of Psychiatry and Human Behavior, Brown University Medical School, Providence, USA; School of Medicine, Department of Psychiatry, University of California, San Diego, La Jolla, USA; Division of Academic General Pediatrics, Developmental Pediatrics, and Community Health, University of California, San Diego, School of Medicine, 9500 Gilman Drive, MC 0874, La Jolla, CA 92093 USA

**Keywords:** General parenting, Parenting style, Parent behaviors, Childhood obesity, Family meals, Measurement, Assessment

## Abstract

**Background:**

There is growing interest in the relationship between general parenting and childhood obesity. However, assessing general parenting via surveys can be difficult due to issues with self-report and differences in the underlying constructs being measured. As a result, different aspects of parenting have been associated with obesity risk. We developed a more objective tool to assess general parenting by using observational methods during a mealtime interaction.

**Methods:**

The General Parenting Observational Scale (GPOS) was based on prior work of Baumrind, Maccoby and Martin, Barber, and Slater and Power. Ten dimensions of parenting were included; 4 were classified in the emotional dimension of parenting (*warmth and affection, support and sensitivity, negative affect, detachment)*, and 6 were classified in the behavioral dimension of parenting (*firm discipline and structure, demands for maturity, psychological control, physical control, permissiveness, neglect)*. Overweight children age 8–12 years old and their parent (n = 44 dyads) entering a weight control program were videotaped eating a family meal. Parents were coded for their general parenting behaviors. The Mealtime Family Interaction Coding System (MICS) and several self-report measures of general parenting were also used to assess the parent–child interaction. Spearman’s correlations were used to assess correlation between measures.

**Results:**

The emotional dimensions of warmth/affection and support/sensitivity, and the behavioral dimension of firm discipline/structure were robustly captured during the family meals. Warmth/affection and support/sensitivity were significantly correlated with affect management, interpersonal involvement, and communication from the MICS. Firm discipline/structure was inversely correlated with affect management, behavior control, and task accomplishment. Parents who were older, with higher educational status, and lower BMIs were more likely to display warmth/affection and support/sensitivity.

**Conclusion:**

Several general parenting dimensions from the GPOS were highly correlated with similar family functioning constructs from the MICS. This new observational tool appears to be a valid means of assessing general parenting behaviors during mealtimes and adds to our ability to measure parent-level factors affecting child weight-related outcomes. Future evaluation of this tool in a broader range of the population and other family settings should be conducted.

## Background

There has been long-standing interest in the influence of general parenting on many aspects of child development. In more recent years, there has been particular interest in its relationship to childhood obesity [1]. However, measuring general parenting styles can be difficult, partly due to differences between measures regarding underlying constructs, level of parenting behavior being measured, psychometric properties of the measures, and participants’ understanding of individual assessment items [2,3]. This variability has resulted in inconsistencies regarding the relationship between parenting and childhood obesity [4-7]. As a result, the American Heart Association and the International Society for Behavioral Nutrition and Physical Activity have called for better measures of parenting to help inform the impact of general parenting on weight related behaviors and ultimately childhood obesity [2,8].

**Figure 1:**
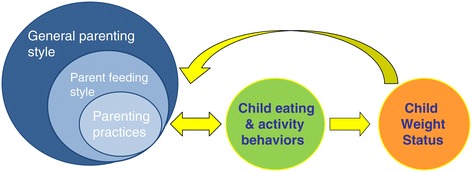
**Parent levels of influence on child behaviors and weight.** There are three levels of parenting that have been identified as impacting child eating and activity behaviors and weight status. The broadest is general parenting style, which is thought to moderate the effect of specific parenting practices and possibly parent feeding style. Each of these levels of parenting can influence child eating and activity behaviors and ultimately child weight status, either individually or in conjunction with each other. Child eating and activity behaviors directly affect child weight status. This relationship however seems to be bi-directional, and child weight status and eating and activity behaviors influence parenting behaviors.

Within the domain of parent feeding and child nutrition, there are three different levels of parenting that have been reported on in the literature: specific parenting practices, parent feeding style, and general parenting (Figure 1) [9]. General parenting is the broadest concept of parenting and is traditionally thought of as the underlying attitude and socialization goal parents have towards their children [10]. It often provides the backdrop or emotional context in which specific parenting behaviors are delivered and interpreted by the child. As such, it should not be viewed as what parents do (which is better defined as specific parenting practices), but how they do it. Because it represents an underlying attitude and approach towards parenting however, it can be difficult to measure. For example, a question that is often asked in obesity-related research is whether or not parents limit the amount of food their child eats. This is generally thought of as a question that assesses a specific parenting practice. However, parents may do this by discussing what the appropriate amount is to eat and encouraging children to slow down and not take seconds (authoritative style), or by abruptly telling the child s/he should stop eating and taking the food away after it has been partially consumed (authoritarian style). Depending on how the parent limits food intake (i.e., which general parenting style is used), the impact of the specific parent behavior (in this case, limiting food consumption) on child eating behavior or weight may be altered. This moderating effect of general parenting on specific parenting practices has been demonstrated in a few studies [11-13]. For example, van der Horst and colleagues found that parents were more effective at limiting their child’s consumption of sugar-sweetened beverages when they used an authoritative parenting style compared to when they used an authoritarian parenting style [11]. These studies therefore indicate that higher-order general parenting is an important construct to consider when examining the impact of parent behaviors on child outcomes.

Parent feeding styles reflect the specific attitudes and socialization goals parents have around feeding their child [14], and is another level of parenting that has been examined in relation to pediatric obesity-related outcomes. While the terminology used to describe parent feeding styles are derived from the general parenting literature, this type of parenting is typically thought of as a domain-specific form of parenting (around food) that is nested within the broader concept of general parenting. Because these different levels of parenting have not always been clearly differentiated in the literature, there appear to be inconsistent findings around “parenting” and child obesity-related outcomes [9]. For example, the indulgent/permissive *parent feeding style* has been associated with greater child weight status [15,16] while in other studies, the authoritarian *parenting style* (general parenting) has been associated with greater child weight status [4,17]. As a result, it can be confusing to delineate how parenting relates to child obesity-related outcomes. Nevertheless, because parent feeding styles pertain to a different level of parenting and reflect domain specific goals around child eating, this level of analysis has its own merit and should be considered separately from the higher-order dimension of general parenting.

While specific parenting practices or feeding behaviors often have a stronger relationship with child eating behaviors and weight status than general parenting [18,19], there are several studies to suggest that general parenting style alone is also related to these outcomes [4,20,21]. This is best demonstrated in a recent study that involved a general parenting intervention for children with behavioral problems [22]. The goal of the original intervention was to improve parents’ ability to interact with their children and manage their behavioral problems. While weight loss was not a goal of the original study and nutrition and physical activity counseling was not a component of the intervention, they were still able to demonstrate an effect on obesity rates, with children from the intervention group having lower rates of obesity three to five years later compared to children who did not receive this intervention [22]. Thus it appears that general parenting can have a significant impact on broader aspects of child health and well-being. Understanding the effect of general parenting on these outcomes will help us determine how to incorporate parenting training, specifically which aspect of general parenting training, into obesity prevention and treatment efforts. However, in order to assess this dimension, better measures of general parenting need to be developed.

Because general parenting taps into more abstract parenting concepts like socialization goals and emotional climate, there is an added complexity to assessing it. Contemporary parenting measures have typically relied on self-report or survey items and focus on specific parenting behaviors [1]. Unfortunately, these types of assessments can lead to recall bias and have the added problem of social desirability. Parents may also lack awareness of their own parenting style and behaviors [23]. Others have suggested that using child reports of parenting behavior can result in more relevant accounts since it is ultimately the child’s perspective of his/her parent’s behaviors that influence his/her development [24,25]. However, here again, there are issues with finding developmentally appropriate measures and assessing the views of toddlers or preschool children who are unable to read and write. While there are several newer methods that have the ability to improve the reliability of self-report measures (e.g., using implicit measures, ecological momentary assessment, or computerized adaptive testing [26]), observational methods are a traditional method of assessment that allow for more objective evaluation free from recall bias. Observational methods have their own limitations, such as social desirability, coder biases, and time and labor intensity to analyze. Nevertheless, as interest in the relationship between general parenting and childhood obesity-related behaviors grows, additional methods of assessing general parenting are needed and a return to direct observational methods may be warranted.

### Defining general parenting styles and dimensions

The classic general parenting dimensions include: 1) warmth, support and responsiveness, and 2) behavioral control and demands for maturity [27-29]. The dimension of “warmth, support and responsiveness” is defined by displays of support and emotional connection with the child to foster autonomy and self-efficacy. “Behavioral control/demands for maturity” is characterized by the setting of expectations for the child to display certain levels of maturity and compliance with behavioral norms. Crossing these dimensions result in the four classic parenting styles of authoritative parenting (high in warmth/support and behavioral control/maturity demands), authoritarian parenting (low in warmth/support, high in behavioral control/maturity demands), permissive parenting (high in warmth/support, low in behavioral control/maturity demands) and neglectful parenting (low in warmth/support and behavioral control/maturity demands) [30]. While much of the literature focuses on these four parenting styles, growing attention has been paid to the specific dimensions encompassing these parenting styles. For coding purposes, it may also be simpler and more accurate to assess each of the general parenting dimensions separately. This will allow us to determine specifically which aspects of general parenting are affecting child weight-related outcomes.

### Creation of general parenting observational scale

Based on work by Baumrind [27], Maccoby and Martin [30], Barber [31], and Slater and Power [32], several dimensions of parenting were selected for this direct observational scale. These included traditional dimensions of warmth and support, behavioral control, and demands for maturity, but also included newer concepts like psychological control and structure (Table 1). Similar to how Maccoby and Martin operationalized parenting [30], these were then divided into two categories of parenting, one that characterizes the emotional climate of the parent–child interaction and one that characterizes the behavioral aspects of parenting.

**Table 1:** **Parenting dimensions of the General Parenting Observational Scale**

**GPOS Dimension**	**Definition**
**EMOTIONAL DIMENSIONS**:	
Warmth and affection	Parent expresses warmth and affection towards the child by saying “I love you” or other words of affection, praising the child, or showing that they care about the child. This affection can be reflected in the parent’s tone of voice, facial expressions, physical signs (like hugging, patting on the back, or gentle touching), or other affectionate acts. Parent may also provide positive reinforcement for child behaviors. Overall, parent shows genuine affection, care and attachment towards their child.
Support and sensitivity	Parent provides support and helps the child in some manner. Parent can listen to the child’s ideas; shows physical, emotional, or intellectual support and understanding of the child’s behaviors, thoughts, or emotions; appreciates the child’s ideas and behaviors; helps child to problem solve; and helps child through difficulties. Parent is sensitive to the child’s needs and goals. Ultimately, parent is aware of what the child is doing and adjusting his/her own behavior to take the child’s behaviors and needs into consideration.
Negative Affect	Parent shows anger, hostility, disdain, or disappointment towards the child. Parent may criticize, yell, make fun of child (mocking), belittle, make sarcastic comments towards child, or be frustrated by what the child is saying or doing. This attitude can be reflected in the tone of voice, facial expressions, or hostile acts.
Detachment	Parent is uninvolved or unresponsive towards the child. For example, the child may do something nice for the parent, but the parent does not acknowledge it. Parent can be distant or is “going through the motions”, but displays no feeling of attachment with the child. There is an overall lack of connection with child. Parent may be actively ignoring the child (e.g. child is trying to interact or get the parent’s attention but is not getting a response, or the child is being “boxed” out of conversation/interaction).
**BEHAVIORAL DIMENSIONS**:	
Firm discipline and structure	This dimension captures how parents structure the environment to control or manage the child’s behaviors. Parents have adefined set of rules, guidelines, and boundaries for behaviors that are somehow expressed on the recording. For example,parent may enforce or remind the child about a rule or expectation, explain reasons for a rule, allow discussion around arule, provide warnings, or carry through with some disciplinary action or consequence. Parent may demonstrate flexibilityaround certain rules but usually has a limit which is not negotiable. Parent tries to be consistent when disciplining andcarry through with the discipline or consequence. He/she expects the child to follow rules and structures the environmentto support these behaviors.
	(Parents can be calm or angry when disciplining, but if they are angry, using threats, raising their voice, or bullying, then also code for *negative affect.)*
Demands for maturity	Parent expects certain behaviors from the child that demonstrate maturity and respect for others, like not interrupting, saying please and thank you, using a napkin or silverware appropriately, etc. Parent also expects self-control of behaviors, emotions, and attitudes. Parents may remind the child of these expectations verbally or refer to these expectations through physical acts, gestures, or facial expressions.
Psychological control	This type of control intrudes into the psychological and emotional development of the child, and typically includes guilt or coercion to influence the child’s behaviors (guilt induction). Parents can show disappointment in the child behaviors or tell the child about all the sacrifices that were made for the child with the intention of guilting or persuading him/her to execute or complete the desired behavior. Parent may bring up previous bad behavior as a reminder to influence a new behavior. Parent may also withdraw affection if the child does something bad (love withdrawal), invalidate the child’s feelings, make a personal attack on the child, and demonstrate erratic emotional behavior (change their emotional reaction to suit their needs and goals). Parent can also be intrusive and push his/her goals and agenda on the child without regard for what the child is doing. Parent typically wants to control all of the child’s behaviors and wants to tell the child what to do. Child has no autonomy in this situation.
Physical control	Parent uses physical force to control the child’s behavior. Parent may physically hurt the child, push or grab the child, or spank the child when he/she disobeys.
Permissive	In this situation, the child usually decides what to do and controls his/her behaviors, actions, and daily schedule. The child can also determine the rules, e.g. what to eat, how much to eat. There are typically no rules. Parents are more laissez-faire. They may label the child’s misbehavior, but provide no follow-through with discipline. Parents may be more concerned with the child liking them and are therefore not as concerned about the discipline. These parents usually cannot say no to the child.
Neglectful	Parent does not provide support or respond to the child’s physical needs. For example, if the child hurts him- or herself, parent does not respond or show concern; or the parent does not provide more food or drink if the child asks for it or looks hungry. This is different from *detachment* in that it does not address the emotional needs of the child.

Dimensions were based on classic parenting concepts introduced by Baumrind, Maccoby and Martin, Barber, and Slater and Power.

Videotapes are divided into 2 minute time-periods and each dimension is scored on a scale from 1 (not at all present) to 5 (present a great deal). Composite scores are calculated for each dimension based on a 20 minute videotape of a family meal.

#### Emotional dimensions of parenting

The emotional dimensions of parenting included warmth and affection, support and sensitivity, negative affect, and detachment. *Warmth and affection* was rated on how often the index parent demonstrated love, caring and affection for the index child, either verbally, physically, or through facial expressions. *Support and sensitivity* was rated on how engaged the parent was with what the child was saying or doing, demonstrating sensitivity to his/her needs and providing understanding and support for the child’s behaviors, thoughts, and emotional expressions. *Negative affect* was scored when parents demonstrated hostility and anger towards the child. Sarcastic remarks were also included in this category. Parents were scored for *detachment* if they were unresponsive towards the child and displayed any behavioral expression to suggest a lack of feeling of attachment or interest in what the child was feeling, saying or doing.

#### Behavioral dimensions of parenting

The behavioral dimensions of parenting included firm discipline and structure, demands for maturity, psychological control, physical control, permissiveness, and neglect. *Firm discipline and structure* was rated on how often parents limited behaviors and enforced rules. Consistency in enforcing these rules contributed to scoring in this dimension. Because structure complements the concept of firm discipline, it was coded together. *Structure* is defined as having clear rules and routines, consistent boundaries, and an organized environment in which the child can exist [32]. Parenting that does not include structure does not provide an organized environment in which the child can learn what their parent’s behavioral expectations are. The organized home environment and consistent parenting provided by structure ultimately provides the child with stable expectations and an ability to develop and learn new skills successfully [33]. Recently, this dimension of general parenting has appeared in a comprehensive self-report measure of parenting [34] and is garnering more interest in the field. The use of routines, reference to rules that had previously been set, or the discussion of new rules were included when scoring for firm discipline and structure. *Demands for maturity* is a behavioral dimension that compliments that of firm discipline and structure, and refers to the behavioral expectation that parents have for their child to demonstrate self-control and maturity. Parents who remind their children of these behavioral expectations were coded as displaying demands for maturity. If any of these demands were delivered with negative affect, coders would include that rating in the appropriate emotional dimension of parenting.

In addition to these dimensions, *psychological control* was included. Psychological control is different from behavioral control in that it uses more coercive and emotionally-laden parenting behaviors (e.g., guilt induction, withdrawal of love, disappointment, shame) as well as excessive use of personal control (e.g., possessiveness and protection) to manage a child’s behaviors [31]. Parents who use this more intrusive type of parenting often impose their agenda onto the child, reflecting a more domineering type of control over the child’s autonomy. This type of parenting can have a negative impact on child social and emotional growth and development into an autonomous, independent, and self-efficacious person. It has also been shown to have a negative impact on adolescent substance abuse [35], internalizing problems, and self-efficacy [19]. More recently, it was found to be associated with higher child BMI z-scores [36] and was therefore included in this scale. Psychological control was rated on whether the parent used coercive behaviors like guilt induction or withdrawal of love and attention to control or shape the child’s behaviors. Parents who were intrusive, or showed little regard for their child’s interests or agenda, were rated highly on this dimension.

Other aspects of behavioral parenting included *physical control*, *permissiveness*, and *neglect. Physical control* was intended to capture any physical actions that the parent used to control the child’s behavior. *Permissiveness* was scored when parents showed little control over their child’s behaviors and allowed the child to determine the rules. Parents were also rated as being permissive if they did not follow through with any suggestion of discipline or had a hard time placing limits on the child’s behaviors. *Neglect* was included to capture parents who were not responsive to the child’s physical needs. This was meant to be different from detachment in that detachment pertained to whether or not the parent addressed the child’s emotional needs and displayed a level of emotional attachment with the child. A parent could be detached emotionally, but still meet the child’s physical needs, and therefore not be neglectful.

With this new observational schema, we set out to assess general parenting dimensions during a family meal. While other parenting coding schemas use the play-situation [37,38], this setting may not be developmentally appropriate for older children. Mealtimes offer a naturalistic setting where parents are often managing child behaviors, imposing rules and expectations, and interacting with their children. Furthermore, it allows one to assess other parent level behaviors, like feeding style and feeding practices, as well as family functioning that may be associated with obesity-related outcomes. Several behavioral observational measures of parent/caregiver-child interactions during mealtime exist [39-44]. While one of these measures claims to assess maternal parenting style, closer examination of this tool reveals that it measures feeding specific behaviors instead (e.g., prompts to eat and number of times food is offered) [39]. Several of the other tools also report on feeding-specific parenting behaviors or prompts that occur prior to eating [40,42,43]. Finally, one tool characterizes caregiver feeding behaviors into the four classic parenting styles, thus capturing parent feeding styles and not general parenting per se [44]. As such, very few observational tools truly assess the higher-order level of general parenting. The purpose of this study was to develop and test a tool to assess general parenting as it pertains to obesity-related outcomes. The Mealtime Family Interaction Coding System (MICS) has been used in previous studies to assess family functioning in homes with overweight children [45,46] and several of its dimensions capture similar emotional and behavioral control aspects of parenting, albeit at a different level of interaction. Therefore, we used the MICS and several self-report measures of parenting to examine the correlation between their constructs of parenting to those from our newly developed General Parenting Observational Scale (GPOS).

## Methods

### Subjects

Families were recruited through advertisements in physicians’ offices and schools in Providence, Rhode Island and San Diego, California. Families with overweight (body mass index (BMI) ≥ 85^th^ percentile but < 95^th^ percentile) or obese (BMI ≥ 95^th^ percentile) children were recruited to participate in a family-based weight control intervention for children between the ages of 8 and 12 years old. Eligible families had a child with a BMI ≥ 85^th^ percentile but were less than 100% overweight, and were willing to participate in the 16-week program. Families were excluded if the child was taking medication that affected his/her weight or growth, was severely developmentally delayed, had a major psychiatric illness that prevented him/her from participating in the group sessions, or they were moving outside the area during the timeframe of the study. Families were provided with informed consent and IRB approval was obtained from relevant institutions in both cities.

### Procedure

Families who contacted the research center completed a brief phone screen. Those who were eligible were invited to the center for an orientation session where they learned more about the weight control intervention. Families with overweight children were invited to participate in a 16-week family-based weight control program based on the Traffic Light Diet [47,48]. Children and parents met in separate 1-hour weekly group sessions and learned about behavioral strategies that would help them make changes to their eating and physical activity behaviors. A total of 226 parents contacted the research center. However, 182 families were excluded because of failure to meet eligibility criteria (19%), lack of interest or time commitment (54%), did not respond to phone calls or attend orientation (21%), or failure to complete pre-intervention assessments (6%). As a result, only 44 families entered the study. Pre- and post-intervention assessments were completed by both children and parents. Only pre-intervention assessment data from families were used in this study.

Each family participated in a video-taped family meal before and after the intervention. Research assistants (RA) scheduled a time to go to the family’s home during one of their regular dinner hours (times ranged from 3:30 to 7:30 P.M.). The RA set up a video camera in their dining area and turned the camera on when they were ready to eat. She then left the house and returned in 30 minutes. Since previous studies have demonstrated that parent–child behaviors during a meal are similar across three different tapings [49-51], only one taping was performed for each family. However, to ensure the validity of this taping, after the meal parents were asked to rate: 1) how similar the meal was to their typical meal, and 2) how similar the parent–child interaction was to their typical interactions. Scores were rated on a scale of 1 (not very typical) to 4 (very typical). Parents who scored the meal or the interaction as a 1 or 2 were told that an additional meal would have to be taped to ensure the validity of the data being collected. Only 2 families required an additional taping.

### Measures

#### General Parenting Observational Scale (GPOS)

Based on the Home Observation Coding System [52], a 5-point global rating scale was used to determine the prevalence of these general parenting dimensions during the meal. Coding began when all family members were at the table. Meals averaged 18.1 minutes (S.D. 3.2 minutes) in length (median = 20 minutes, interquartile range = 4 minutes). Therefore tapes were coded for 20 minutes. Each 20-minute videotaped family meal was divided into 10, two-minute time-periods. At the end of each 2 minute segment, the tape was stopped and coders scored the index parent (i.e., the parent who enrolled and came to the intervention) and child interaction for each of the 10 parenting dimensions. Scores ranged from 1 (not present at all) to 5 (present a great deal). Scores were summed for each dimension with a possible range of 10 to 50. Meals less than 20 minutes long were coded and summary scores standardized to fit a 20 minute coding period.

Two coders were trained on the Global Parenting Observational Scale. Coders were educated on the different parenting dimensions included in the scale. Education included coding of ten training tapes in a group setting so that coders could learn to differentiate between the different dimensions. Coders then individually coded “gold-standard” training tapes until they reached at least 70% reliability on these tapes. When they reached this goal, they began coding study tapes. Coders met weekly with the trainer and coded 2 training tapes together to prevent observer drift. All tapes were reviewed by both coders and reliability checked. Inter-rater reliability was determined by performing intraclass correlations between the summary scores for each of the 10 parenting dimensions. Intraclass correlations for each dimension were: warmth/affection = 0.87, support/sensitivity = 0.89, negative affect = 0.91, detachment = 0.81, firm discipline/structure = 0.87, demands for maturity = 0.91, psychological control = 0.95, physical control = 0.96, permissiveness = 0.85, and neglect = 0.74. Coders reached consensus on any discrepant scores and these scores were used for analysis.

### Mealtime Family Interaction Coding System (MICS)

The MICS is an observational coding system based on the McMaster Model of Family Functioning [53,54] and was adapted from the McMaster Structured Interview of Family Functioning. The MICS demonstrates moderate to high correlation with other measures of parent and family functioning [55] and codes six dimensions of family functioning: Task accomplishment, Communication, Affect management, Interpersonal involvement, Behavioral control, and Roles. Overall Family Functioning is the seventh and final dimension that is rated and is not an average of the other six dimensions. Instead, it provides an overall assessment of the quality of the family’s interactions and functioning at the meal. Given the similarity of some of these dimensions with the GPOS parenting dimensions of warmth/affection, support/sensitivity, firm discipline/structure, and demands for maturity, we compared constructs between both measures.

*Affect management* and *interpersonal involvement* assess the emotional aspects of the family meal. *Affect management* addresses the appropriateness and intensity of the emotions expressed at the meal as well as the responsiveness and sensitivity of these emotional responses towards other family members. *Interpersonal involvement* captures the degree to which family members show respect, interest, and value in each other’s activities and thoughts (similar to support and sensitivity in the GPOS). *Behavior control* assesses the way in which the family maintains rules around physical expectations at the table and social behaviors. It categorizes the behaviors as chaotic, laissez-faire, rigid, and flexible, and is similar in part to the parenting styles of permissive, authoritarian, and authoritative. Families who shift between different control styles and use more chaotic, rigid, or laissez-faire methods score lower in this domain. *Task accomplishment* also assesses the structure and organization of the meal and reflects on the parent’s ability to have control over the meal. Families who display smooth transitions between tasks and can adequately handle disruptions score higher in this domain. *Communication* rates the verbal interaction of the family, particularly the rate of exchange of information, the quality of the communication, and the appropriateness between different age groups. Finally, *roles* reflect on the patterns of behavior of each family member and whether or not they are able to fulfill expected tasks. Each dimension is rated on a Likert scale from 1 to 7, with scores of 5 or greater being considered categorically different (healthy) from those with scores less than 5 (unhealthy).

Coding for the MICS was performed by independent trained coders who were blind to study hypotheses.

### Child measures

Child’s Report of Parental Behavior Inventory (CRPBI) [56] asks children to assess their parent’s parenting behaviors [57], and can be completed by children aged eight years and older [58]. It has been used in pediatric weight control studies [59], as well as adapted to assess parent involvement and strictness in relation to dietary [20,21] and smoking behavior [60]. The inventory assesses three dimensions of parenting: acceptance vs. rejection, psychological control vs. autonomy, and firm vs. lax control. The 30 item version (CRPBI-30 [61]), which is a shortened version of the 108-item scale [62], was used in this study and includes the top ten items with the highest correlation within each dimension. Children rated each item on a 3-point Likert scale ranging from “like”, “somewhat like”, or “not like” their parent’s behavior. Children completed this measure for their mother and father’s parenting behaviors separately. Factor analysis demonstrated that each of the items loaded significantly on a single principle axis with 96%, 94%, and 87% of the variance respectively. The alpha values for Acceptance, Psychological control, and Firm control have been previously reported as 0.75-0.73, 0.72-0.63, and 0.65-0.63 respectively [61]. The test-retest correlations ranged from 0.79-0.89. This inventory has been reported to have strong discriminative validity [63].

*“Getting Along with My Parent”* [64] is a 38-item questionnaire (19 items relating to the mother and 19 items for the father) that assesses the child’s ratings of the caregiver’s behaviors. The items map onto 2 dimensions, warmth/support and hostility. Examples of the warmth/support items include: “When you and your mother spend time talking or doing things together, how often does your mother: let you know she really cares about you; listens carefully to your point of view; acts supportive and understanding towards you; helps you do something that is important to you?” Examples of the hostility items include: “When you and your mother spend time talking or doing things together, how often does your mother: get angry at you; criticize your ideas; shout or yell because she is mad at you; insult or swear at you?” Four-point scales were used to assess parent behaviors from “a lot” to “not at all”. The items have been previously reported to have an internal consistency of 0.78 for the warmth scale and 0.79 for the hostility scale [65].

### Parent measures

*Parent Report of Parental Behavior Inventory (PRPBI)* is a 30-item measure that parallels the CRPBI-30 and was used to assess parent’s views of their own parenting behaviors towards the index child. This measure assesses the same three dimensions of parenting as the CRPBI, has the same scoring system, and has been used successfully in previous studies [66,67].

*Raising Children Checklist (RCC)* [68] is a simplified revision of Greenberger’s Raising Children Checklist [69], a standardized measure of parenting strategies that was based on Baumrind’s concepts of responsiveness and disciplinary control. This measure has been used in NICHD’s Study of Early Child Care and Youth Development [70] and was chosen for its brevity and apparent face validity with Baumrind’s dimensions of parenting. Three dimensions of parenting are obtained with this survey – firm (authoritative), harsh (authoritarian), and lax (permissive) parenting. These dimensions have been found to differentiate children based on school adjustment, academic achievement, and behavioral problems [69]. Cronbach’s alpha for each of these dimensions were reported as 0.67, 0.75, and 0.73 respectively.

#### Anthropometrics

Child height and weight were obtained to determine BMI percentile and BMI z-scores. Weight was measured in kilograms to the nearest 0.1 kg on a Tanita Digital Scale (model WB-110A). Weight was measured twice and the average of the values was used for analysis. Height was measured using a portable Tanita stadiometer. Height was recorded to the nearest 0.1 cm for both trials, and the average of the 2 values used for analysis. Body mass index (BMI = [kg/m2]) was calculated and translated to BMI percentiles for age and sex using the CDC growth charts [71] and to standardized BMI z-scores (BMI-Z ) [72].

Several sociodemographic variables were included in this study: parent and child age and gender, parent race/ethnicity, marital status and educational level. In this sample, the primary racial/ethnic groups were Caucasian, Hispanic, and other. Maternal education was dichotomized into “some college or less” and “college degree or higher”. Marital status was dichotomized into “married or living with significant other” and “widowed, divorced, separated or never married”.

#### Analysis

Descriptive statistics and correlations were completed using SAS, version 9.3. Distributions of the GPOS dimensions were not normally distributed for all dimensions except warmth/affection and support/sensitivity. As a result, medians and interquartile ranges were presented. Spearman’s correlations were used to determine correlations between the GPOS parenting dimensions and the MICS family functioning dimensions, child reports of parenting behaviors, and parent reports of parenting behaviors. Correlations between parenting dimensions and parent demographics were also explored. Alpha level of 0.05 was used to determine significance.

## Results

The sample included 44 parent–child dyads. Mean child age was 10.0 years and two-thirds were female. Mean child BMI percentile was 98.2. The majority of parents were mothers with a mean age of 41.4 years. Most children lived in two parent households and about half of parents had a college degree or higher (Table 2). There were no significant differences in demographic characteristics between those families recruited in Rhode Island and California.

**Table 2:** **Child and parent demographics**

**Variable**	**Percent or Means (S.D.) (n = 44)**
**Child characteristics**	
Sex	
Male	34%
Female	66%
Age (years)	10.0 (1.3)
BMI percentile	98.2 (1.3)
BMI z-score	2.2 (0.3)
**Parent characteristics**	
Sex	
Male	5%
Female	95%
Race/ethnicity	
White	52%
Hispanic	36%
Other	11%
Education	
No degree	47%
Bachelor’s degree or higher	53%
Marital status	
Married/living with significant other	77%
Widowed/divorced/Separated/never married	23%
Age (years)	41.4 (6.9)
BMI (kg/m^2^)	30.1 (5.8)

S.D. = Standard deviation.

Scores for the observed parenting dimensions ranged between 10 and 34 (Table 3). Only five dimensions had a wide range of scores: warmth/affection, support/sensitivity, negative affect, detachment, and firm discipline/structure. Of these dimensions, the most commonly observed behaviors were warmth/affection, support/sensitivity, and firm discipline/structure. All other parenting dimensions were not commonly observed during the videotaped family meals.

**Table 3:** **Range of observed parenting dimensions in the General Parenting Observational Scale**

	**Mean (S.D.)**	**Median (IQR)**	**Range**
**Emotional Dimensions**			
Warmth and Affection	23.70 (6.49)	24.0 (9.5)	10-34
Support and Sensitivity	23.37 (5.28)	24.44 (6.43)	10-33
Negative Affect	10.99 (3.18)	10.0 (1.0)	10-30
Detachment	10.99 (3.42)	10.0 (0)	10-30
**Behavioral Dimensions**			
Firm discipline/ structure	12.95 (3.64)	12.0 (4.44)	10-29
Demands for maturity	10.52 (0.73)	10.0 (1.0)	10-13
Psychological control	10.09 (0.59)	10.0 (0)	10-14
Physical Control	10.05 (0.34)	10.0 (0)	10-12
Permissive	10.38 (1.14)	10.0 (0)	10-16
Neglect	10.13 (0.63)	10.0 (0)	10-14

Tapes were coded for 20 minutes. Tapes were divided into 10 two-minute intervals and coded for all 10 parenting dimensions. Summary scores for each dimension ranged from 10–50.

S.D. = Standard deviation.

IQR = Inter-Quartile Range.

Several parenting dimensions were highly correlated with the family functioning dimensions of the MICS (Table 4). Families who scored highly in warmth/affection (GPOS) scored highly in affect management, interpersonal involvement, and communication (MICS) (r = 0.39, 0.56, and 0.44, respectively). Support/sensitivity (GPOS) was also highly correlated with affect management, interpersonal involvement, and communication (MICS) (r = 0.38, 0.55, and 0.45, respectively). High scores in negative affect (GPOS) were inversely correlated with affect management, interpersonal involvement, communication, behavior control and overall family functioning (MICS). In the behavioral dimensions, there was an inverse relationship between firm discipline/structure (GPOS) and the family functioning dimensions of affect management, behavior control, task accomplishment, and roles. Higher permissive scores in the GPOS were also negatively correlated with these dimensions.

**Table 4:** **Correlation between General Parenting Observational Scale and other measures of parenting and family functioning**

	**Warmth/ Affection**	**Support/ Sensitivity**	**Negative Affect**	**Detachment**	**Firm Discipline/ Structure**	**Maturity Demands**	**Psychological Control**	**Physical Control**	**Permissiveness**	**Neglect**
**MICS**:										
Task Accomplishment	0.19	0.15	−0.20	−0.09	−0.31	−0.07	−0.28	−0.28	−0.36	−0.15
	0.23	0.34	0.20	0.59	0.04	0.66	0.06	0.06	0.04	0.32
Communication	0.44	0.45	−0.40	−0.13	−0.23	0.16	−0.27	−0.27	−0.20	−0.31
	<0.01	<0.01	<0.01	0.39	0.13	0.31	0.08	0.08	0.19	0.05
Affect Management	0.39	0.38	−0.44	−0.31	−0.32	0.04	−0.28	−0.28	−0.41	−0.39
	0.01	0.01	<0.01	0.04	<0.03	0.81	0.07	0.07	<0.01	0.01
Interpersonal Involvement	0.56	0.55	−0.42	−0.14	−0.25	0.03	−0.27	−0.27	−0.38	−0.30
	<0.001	<0.001	<0.01	0.36	0.11	0.84	0.08	0.08	0.01	0.05
Behavior Control	0.31	0.27	−0.30	−0.24	−0.35	0.07	−0.29	−0.29	−0.47	−0.38
	0.05	0.08	0.05	0.12	0.02	0.64	0.07	0.07	0.001	0.01
Roles	0.50	0.56	−0.37	−0.32	−0.24	0.15	−0.29	−0.29	−0.37	−0.22
	<0.001	<0.001	0.01	.04	0.12	0.34	0.06	0.06	0.01	0.17
Family Functioning	0.48	0.51	−0.40	−0.26	−0.34	−0.03	−0.27	−0.27	−0.38	−0.30
	<0.01	<0.001	<0.01	0.09	0.02	0.85	0.08	0.08	0.01	0.05
**Getting along with my parent**:										
Warmth	−0.25	−0.29	−0.06	−0.41	−0.12	−0.19	−0.21	−0.21	0.09	−0.05
	0.12	0.06	0.70	<0.01	0.46	0.24	0.18	0.18	0.58	0.75
Hostility	0.05	0.11	−0.19	−0.26	0.00	0.06	0.01	0.01	−0.002	−0.18
	0.75	0.49	0.22	0.10	0.99	0.70	0.93	0.93	0.99	0.27
**CRPBI**:										
Acceptance	0.19	0.05	−0.22	0.13	−0.14	0.08	−0.19	−0.19	−0.23	−0.25
Psychological Control	0.24	0.77	0.17	0.41	0.39	0.61	0.24	0.24	0.14	0.12
	−0.15	0.06	−0.11	−0.15	−0.01	−0.22	−0.24	−0.24	−0.04	−0.30
	0.35	0.73	0.50	0.37	0.94	0.16	0.13	0.13	0.79	0.06
Firm Control	−0.08	−0.00	0.08	−0.09	0.12	−0.14	0.04	0.04	−0.05	0.17
	0.62	0.99	0.61	0.57	0.44	0.38	0.83	0.83	0.77	0.30
**PRPBI**:										
Acceptance	−0.18	−0.21	0.04	−0.01	0.06	−0.06	−0.20	−0.20	0.06	−0.11
	0.26	0.18	0.80	0.96	0.70	0.73	0.21	0.21	0.71	0.49
Psychological Control	−0.45	−0.38	0.25	0.28	0.32	0.00	0.27	0.27	0.36	0.16
	<0.01	0.01	0.11	0.08	0.04	0.98	0.09	0.09	0.02	0.31
Firm Control	−0.52	−0.48	0.19	0.25	0.25	−0.18	0.07	0.07	0.34	0.10
	<0.001	0.001	0.22	0.11	0.11	0.27	0.65	0.65	0.03	0.52
**Raising Children Checklist**										
Harsh	−0.30	−0.22	0.02	−0.11	0.05	−0.20	−0.04	−0.04	−0.05	−0.20
	0.05	0.16	0.88	0.49	0.73	0.21	0.81	0.81	0.77	0.20
Firm	0.05	0.09	−0.27	−0.15	−0.30	−0.09	−0.24	−0.24	−0.29	−0.29
	0.75	0.56	0.09	0.33	0.05	0.56	0.13	0.13	0.07	0.06
Lax	0.11	0.11	0.08	0.27	0.36	0.29	0.01	0.01	0.27	0.18
	0.48	0.48	0.60	0.09	0.02	0.07	0.94	0.94	0.08	0.25

Spearman’s correlation was used to examine the correlation between the 10 dimensions of the GPOS and the MICS and several other child- or self-report measures of parenting. “Getting along with my parent” and the CRPBI are child reports of parenting. The PRPBI and Raising Children Checklist ask parents to report on their parenting. Correlation coefficients (r) and p-values are presented.

MICS = Mealtime Family Interaction Coding System.

CRPBI = Child’s Report of Parental Behavior Inventory.

PRPBI = Parent Report of Parental Behavior Inventory.

When comparing observed parenting dimensions with self-report measures of parenting by children, there were fewer significant correlations. Only child report of warmth (“Getting along with my parent” survey) was inversely correlated with detachment (r = −0.41, p < 0.01). Parent reports of psychological control and firm control (PRPBI) and harsh parenting (Raising Children Checklist) were inversely correlated with observed measures of warmth/affection and support/sensitivity (GPOS). Interestingly, parents self-reporting higher firm (authoritative) parenting (Raising Children Checklist) were viewed as using less firm discipline on the GPOS (r = −0.30). However self-report of lax behaviors was positively correlated with firm discipline on the GPOS (r = 0.36). Similarly, psychological control and firm control (PRPBI) were both positively correlated with permissive behaviors on the GPOS.

Parent demographics were correlated with certain parenting dimensions. Parents with higher BMIs had lower warmth/affection (r = −0.38, p = 0.02) and support/sensitivity scores (r = −0.32, p = 0.05). Older parents had higher warmth/affection (r = 0.33, p = 0.04) and support/sensitivity scores (r = 0.42, p = 0.01). Parents with higher education also had higher warmth/affection scores (r = 0.33, p = 0.04).

## Discussion

The goal of this study was to develop a General Parenting Observational Scale that could be used to assess general parenting dimensions in the context of family meals, and compare these dimensions to those captured in other observational and self-report measures of parenting and family functioning. Parenting dimensions were based on the four classic parenting styles [27,30] and more recent dimensions of interest, i.e., psychological control and structure. The most robust dimensions in the GPOS were warmth/affection, support/sensitivity, and firm discipline/structure. The emotional dimensions of warmth/affection and support/sensitivity were positively associated with the family functioning dimensions assessing interpersonal involvement, affect management, and communication. Thus parents displaying high warmth, affection, and support for their child on the GPOS were also viewed as having good communication, effective and appropriate emotional displays, and being involved with or expressing empathy and concern for their child. Warmth/affection and support/sensitivity were also inversely correlated with parent self-report measures of harsher parenting behaviors. Thus it appears that the emotional dimensions of parenting were captured well in the GPOS.

With regards to the behavioral dimensions, firm discipline/structure in the GPOS was inversely correlated with affect management as well as behavior control and task accomplishment in the MICS. While on the surface this may seem contradictory and questions the validity of this GPOS parenting dimension, these results may actually complement each other and provide us with a more complete picture of what is happening during the family meal. Parents who have to frequently make comments to remind children of the rules and provide a structure for their children so that they comply with these rules (coded as high discipline and structure on the GPOS) may appear to have low behavioral control of the situation on the MICS because of this constant reminding. Furthermore, they may have had to shift between several types of control styles to maintain this order (e.g., chaotic or laissez-faire as defined by the MICS), thus causing them to score low on the MICS. In a similar vein, the frequent reminders about rules may have caused parents to appear as if they were unable to maintain an organized meal with minimal disruptions, resulting in low scores for task accomplishment. The complexity of these family interactions may also explain the results for the GPOS dimension of permissiveness. Parents who were permissive on the GPOS could have had poor behavioral control of the mealtime situation and be unsuccessful at completing the task of eating a meal with minimal disruptions. Thus parents who were permissive would have been categorized as having poor family functioning on the MICS, as was seen in this analysis. These results suggest that the parenting dimensions in the GPOS are related to the concepts captured by the MICS, but not completely overlapping. Overall, the GPOS focuses more on parent behaviors while the MICS takes the whole family and the child’s response to parent behaviors into account. As a result, the GPOS views the behavioral aspect of parenting from another angle, which may potentially complement the results of the MICS. A previous study noted no significant relationship between task accomplishment, behavior control and child BMI z-score [46]. Whether or not the dimensions of firm discipline/structure and permissiveness from the GPOS are related to child BMI remains to be seen. This type of analysis could highlight whether or not the behavioral aspects of parenting captured by the GPOS are providing us with additional information that is not captured in the MICS, thereby providing us with a more complete picture of the parenting and family dynamics during mealtimes.

It is interesting to note that the self-report measures were not as well correlated with the observed parenting behaviors of the GPOS. It was not surprising to see that parents who were viewed as warm/affectionate as well as supportive/sensitive to their child’s needs self-report that they did not engage in psychologically controlling or harsh parenting behaviors. However, observed behaviors of firm discipline, behavioral control, and structure on the GPOS were not self-reported as such. Instead, these parents self-reported more lax parenting behaviors (as measured by the Raising Children’s Checklist). In addition, observed permissive parenting behaviors were self-reported by parents as being more psychologically controlling and firm (PRPBI). When children reported on their parent’s behaviors, only detachment was inversely correlated with parent displays of warmth. There was no correlation between the behavioral dimensions of parenting as assessed by the GPOS or self-report measures. Thus, overall it appears that the emotional dimensions of parenting may be more accurately reported while behavioral dimensions may be subject to personal biases or social desirability. While a few studies have found poor correlations between parent and child reports of parenting behaviors [66,73], we are unaware of any study to date that has compared direct observation of parenting behaviors with parent or child self-report measures of parenting. The results of our study highlight the potential difficulty in using self-report measures of parenting, and suggest that the emotional dimensions of parenting may be more accurately captured in these self-report measures than the behavioral dimensions of parenting.

In our analyses, we also found that parents with higher education, lower BMI scores, and older age scored higher on warmth/affection and support/sensitivity. A few studies using self-report measures of parenting have found that authoritative parenting is associated with higher parental SES [17], and parents with higher levels of education were less likely to use controlling behaviors like coercion or overprotection [34]. Studies have reported that both demographic factors (like higher income and educational attainment [74]) and authoritative or supportive parenting [4] are associated with lower risk for obesity. Whether parenting mediates the relationship between demographic factors and child weight status has not been investigated. Given the correlation between certain parenting behaviors and SES/educational level, this relationship should be clarified and may provide further evidence for targeting general parenting in future interventions.

While several of the dimensions captured in the General Parenting Observational Scale appear to be robust, there were a few limitations. First, the sample was relatively small and only conducted on overweight children and their parents, who were also primarily overweight. Many children also came from two parent families, where general parenting behaviors may be different than in single parent families. Validity of this measure should be tested in larger samples that include a broader range of marital statuses, weight categories, racial/ethnic and cultural groups, and educational backgrounds. Our ability to only record one family meal per subject may have also limited the generalizability of our findings. However other groups have reported that one video recording may be sufficient [49-51], and meals were re-recorded if the parent indicated that the interactions or the meal itself was not typical. Finally, several dimensions were relatively unobserved in this mealtime setting (psychological control, physical control, permissiveness, neglect), and it begs the question as to whether a different parent–child interaction setting should be used to assess a broader range of these general parenting behaviors. Other observational systems, like the Home Observation for Measurement of the Environment (HOME) [37] and Dyadic Parent Child Interaction Coding Scheme (DPICS) [38], have typically been used in play situations. But as children grow older and we assess parenting behaviors at this later time, play situations may not be developmentally appropriate. Given that we are interested in using this scale to determine the relationship between general parenting and childhood obesity and other weight-related behaviors, the mealtime interaction seems to be an appropriate setting. However, we did not videotape and code other eating or activity related situations, like snack time, and the interactions found here may only be relevant to the meal time situation. Examination of this tools’ efficacy in other settings should be explored.

## Conclusion

There is growing interest in the role of general parenting behaviors in the development and potential treatment of childhood obesity. At this time several studies report that authoritative or supportive parenting behaviors are associated with lower risk of obesity [4,5] and improved eating behaviors [20,21]. However, there are still discrepancies reported in these relationships [6,19] and the use of self-report measures of parenting may be contributing to this discrepancy. While there is a role for self-report measures of parenting, particularly when a child reports on his/her parent’s behaviors and this is correlated with his/her developmental outcomes, these measures may still be limited by difficulties with understanding the selected concepts or recall bias. Many people also find it difficult to recognize how their own behaviors impact and are viewed by others. Therefore, an observational tool may provide a more objective or standardized means of measuring parenting. Since there are few observational assessments of general parenting, we developed the General Parenting Observational Scale to assess parent behaviors in a mealtime situation. It appears that the primary dimensions of warmth/affection, support/sensitivity, and firm discipline/structure were robustly captured in this tool. Continued use of this tool among families with a wider age range of child weights and demographic variability will help to determine the versatility of this tool. While observational methods may be more labor and time-intensive, it offers another standardized and possibly more objective means of assessing general parenting. This may result in more homogeneity in research results, allowing one to determine the true relationship between general parenting and obesity-related behaviors.

## Abbreviations

GPOS: General Parenting Observational Scale
MICS: Mealtime Family Interaction Coding System
BMI: Body mass index
RA: Research assistant
CRPBI: Child’s Report of Parental Behavior Inventory
PRPBI: Parent Report of Parental Behavior Inventory
RCC: Raising Children Checklist
BMI-Z: BMI z-score
HOME: Home Observation for Measurement of the Environment
DPICS: Dyadic Parent Child Interaction Coding Scheme
